# Supplementary material for: Chemical logic of MraY inhibition by antibacterial nucleoside natural products
Source: Nat Commun. 2019 Jul 2;10:2917. doi: 10.1038/s41467-019-10957-9 (PMC6606608; doi:10.1038/s41467-019-10957-9)
Supplement: Supplementary file 1 — Supplementary Information [file 41467_2019_10957_MOESM1_ESM.pdf]

Supplementary Information

Chemical logic of MraY inhibition by antibacterial nucleoside natural products

E.H. Mashalidis *et al.*

Supplementary Figures

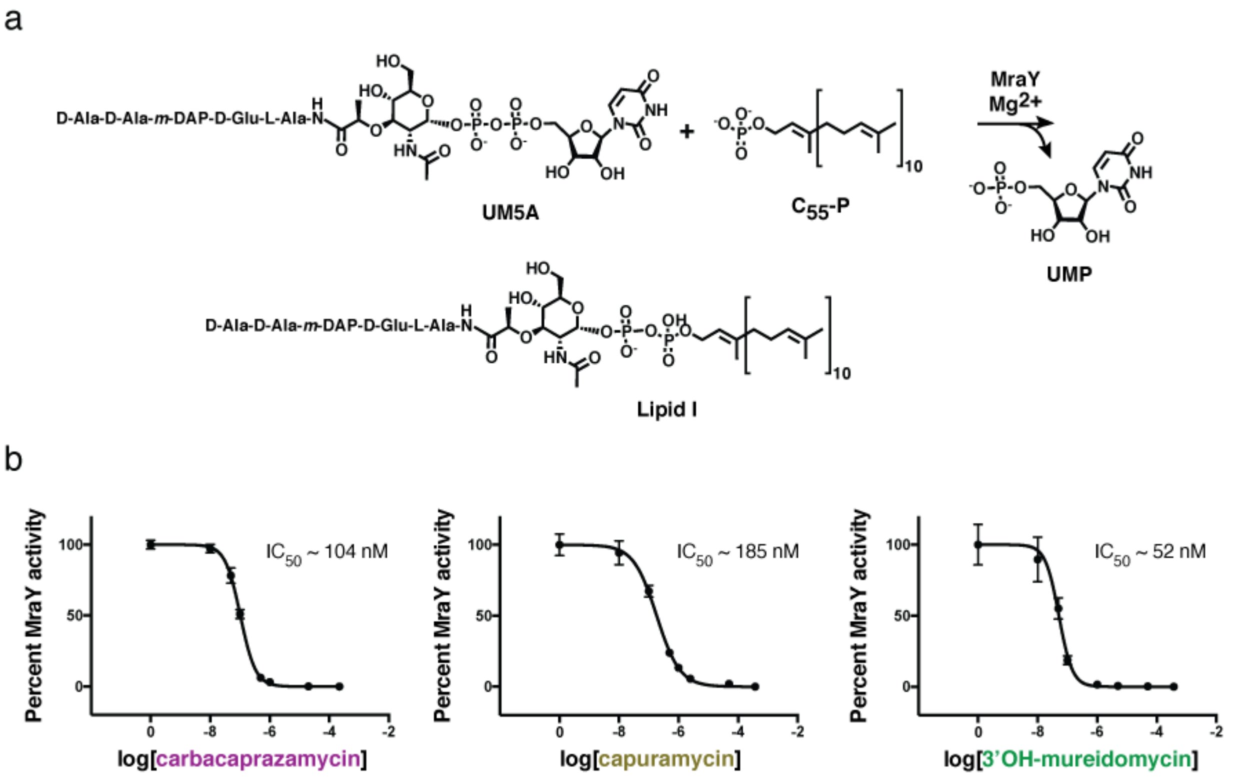

Supplementary Figure 1. $MraY_{AA}$ enzymatic activity is inhibited by carbcaprazamycin, capuramycin, and 3′-hydroxymureidomycin A. (a) Scheme of the MraY-catalyzed reaction. (b) IC_{50} values of carbcaprazamycin, 3′-hydroxymureidomycin A, and capuramycin with $MraY_{AA}$. Each IC_{50} measurement was made using the UMP-Glo™ assay. Data are shown as the mean ± s.e.m. of three technical replicates. The underlying source data for the dose-response curves in (b) are provided in the Source Data file.

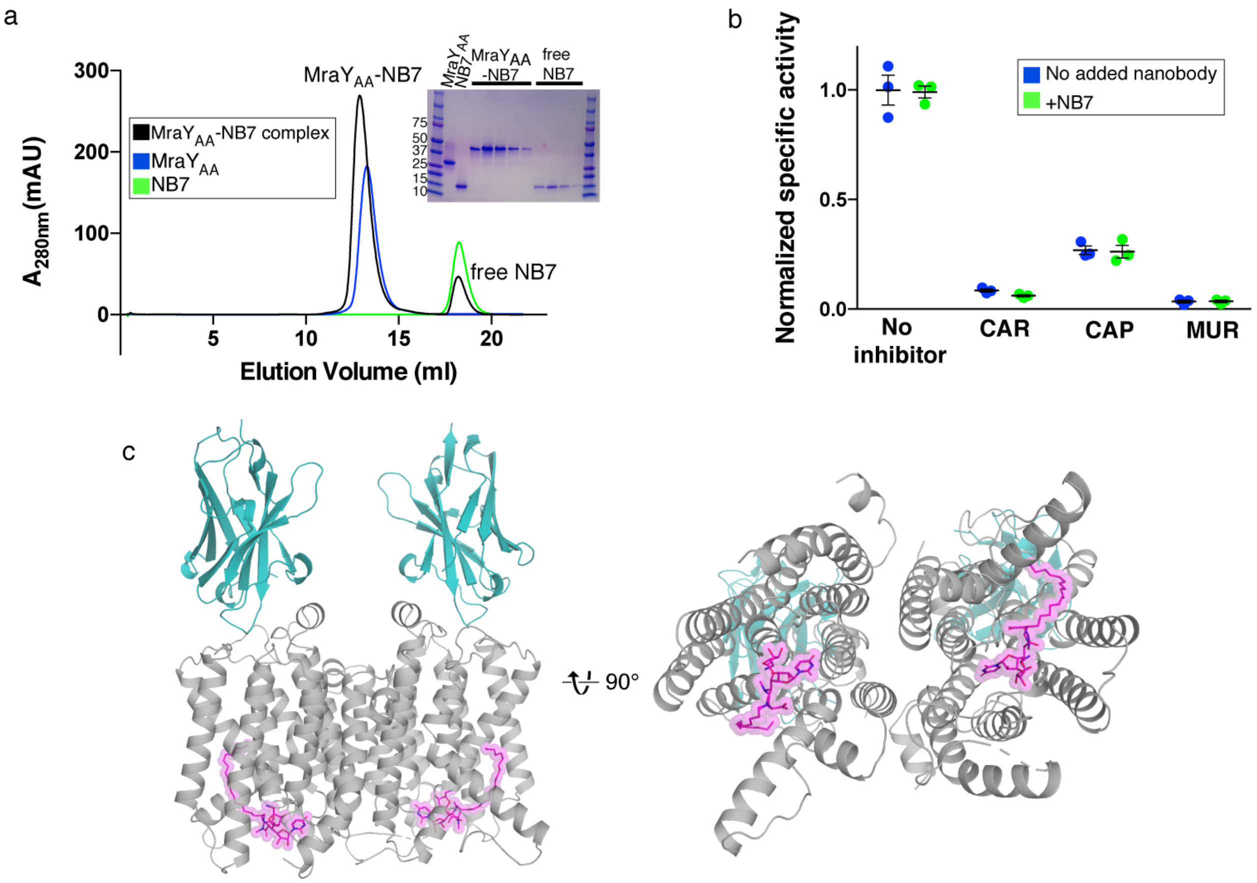

Supplementary Figure 2. MraY$_{AA}$ forms a tight complex with nanobody 7 (NB7), but does not influence MraY$_{AA}$ activity or inhibition. (a) Gel filtration chromatogram and SDS-PAGE analysis of MraY$_{AA}$ (blue), NB7 (green), and the MraY$_{AA}$-NB7 complex (black). To form the complex, MraY$_{AA}$ and NB7 were mixed at a 1:1.5 (MraY$_{AA}$:NB7) molar ratio. The elution volume of the MraY$_{AA}$-NB7 complex is 13.3 mL, while free MraY$_{AA}$ and NB7 elute at 12.9 mL and 18.2 mL, respectively. The leftward shift of the major peak in the MraY$_{AA}$-NB7 mixture sample (black) is due to complex formation, while the minor peak corresponds to the excess NB7. The MraY$_{AA}$-NB7 complex remains intact in SDS-PAGE and runs at around 50 kDa, corresponding to the molecular weight of MraY$_{AA}$ (40.3 kDa) and of NB 7 (13.6 kDa). (b) The presence of NB7 does not affect MraY$_{AA}$ enzymatic activity or inhibition by carbacaprazamycin (CAR), capuramycin (CAP), and 3′-hydroxymureidomycin A (MUR), as demonstrated by specific activity measurements. Each inhibitor was added to a final concentration of 0.5 µM and NB7 was added to a final concentration of 1 µM where present. Specific activity was measured using the UMP-Glo™ assay and each measurement was normalized relative to the no-inhibitor condition. Data are shown as the mean ± s.e.m of three technical replicates. (c) Membrane and cytoplasmic views of the MraY$_{AA}$-NB7 complex structure. NB7 (teal) recognizes the periplasmic face of MraY$_{AA}$ (gray), which is away from the cytosolic carbacaprazamycin (magenta) binding site. The underlying source data for specific activity measurements shown in (b) are provided in the Source Data file.

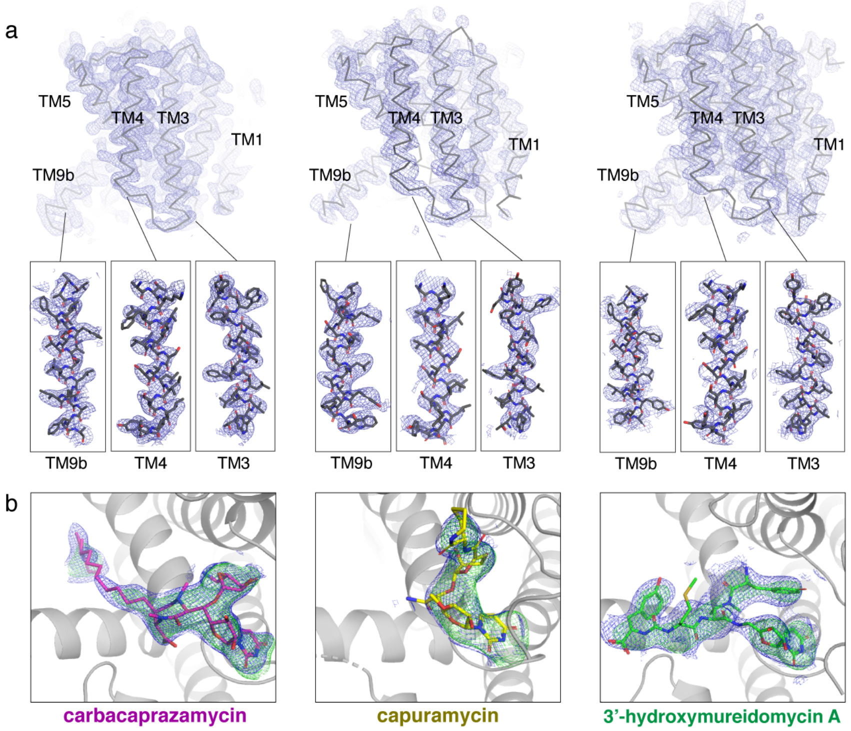

Supplementary Figure 3. Electron density maps for $MraY_{AA}$ in complex with carbacaprazamycin, capuramycin, and 3′-hydroxymureidomycin A. (a) Composite omit maps of $MraY_{AA}$ bound to carbacaprazamycin, capuramycin, and 3′-hydroxymureidomycin A. 2Fo-Fc composite omit maps were calculated for each structure omitting 5% of the model at a time. Maps are shown in slate mesh and contoured to 0.8σ. (b) Omit electron density for carbacaprazamycin, capuramycin, and 3′-hydroxymureidomycin A. The *2Fo-Fc* omit and the *Fo-Fc* omit electron density maps are shown carved around each inhibitor in blue and green mesh, respectively. Carbacaprazamycin, capuramycin, and 3′-hydroxymureidomycin A are shown in magenta, yellow, and green sticks, respectively with *2Fo-Fc* omit electron density contoured to 1.0 σ and *Fo-Fc* omit electron density contoured to 3.0 σ for each.

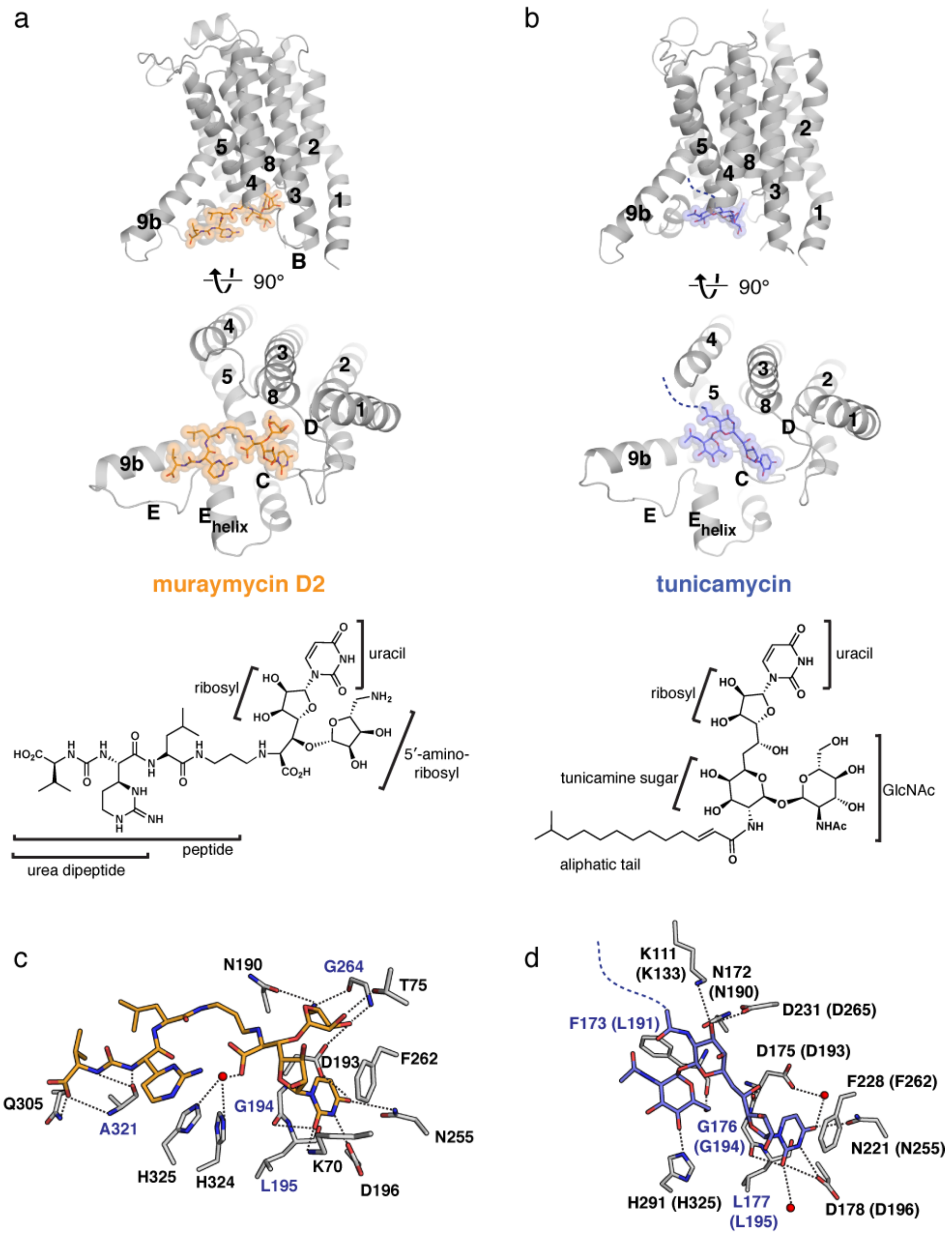

Supplementary Figure 4. Crystal structures of the $MraY_{AA}$-muraymycin and $MraY_{CB}$-tunicamycin complexes. Membrane (top) and cytoplasmic (bottom) views of (a) $MraY_{AA}$ in complex with muraymycin D2 (orange) (PDB ID: 5CKR) and (b) $MraY_{CB}$ in complex with tunicamycin (slate) (PDB ID: 5JNQ). Chemical structures of each inhibitor, with substructures labeled, are shown below the relevant complex structure. Detailed views of interactions formed between (c) $MraY_{AA}$ and muraymycin D2 and (d) $MraY_{CB}$ and tunicamycin. Residue numbering is shown for $MraY_{AA}$ except for in (d), which shows residue numbering for $MraY_{CB}$ with the corresponding residues in $MraY_{AA}$ provided in parentheses. The aliphatic tail of tunicamycin is not resolved and is represented by a dashed line (slate). Hydrogen bonds are shown as black dashed lines and water molecules are shown as red spheres.

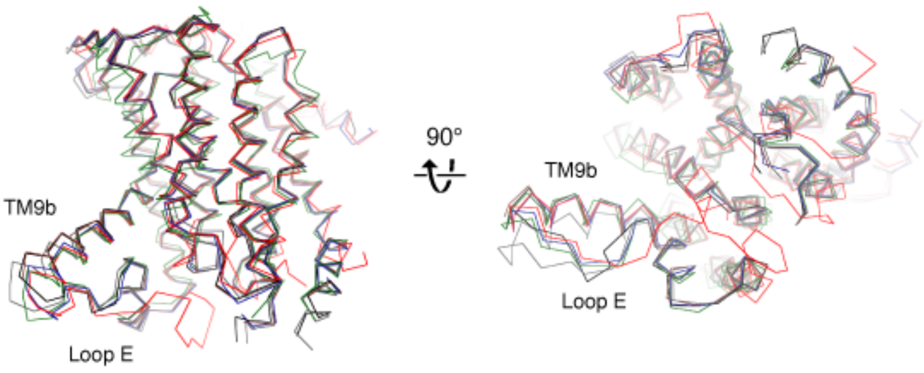

Supplementary Figure 5. Structural variability observed among MraY structures. Structural superposition of $MraY_{AA}$ apoenzyme (red; PDB ID: 4J72) and inhibitor-bound MraY structures: $MraY_{AA}$-carbacaprazamycin (dark blue), $MraY_{AA}$-3′-hydroxymureidomycin A (black), $MraY_{AA}$-capuramycin (brown), $MraY_{AA}$-muraymycin D2 (gray; PDB ID: 5CKR), and $MraY_{CB}$-tunicamycin (dark green; PDB ID: 5JNQ). Protomers are shown for simplicity from membrane (left) and cytoplasmic (right) views.

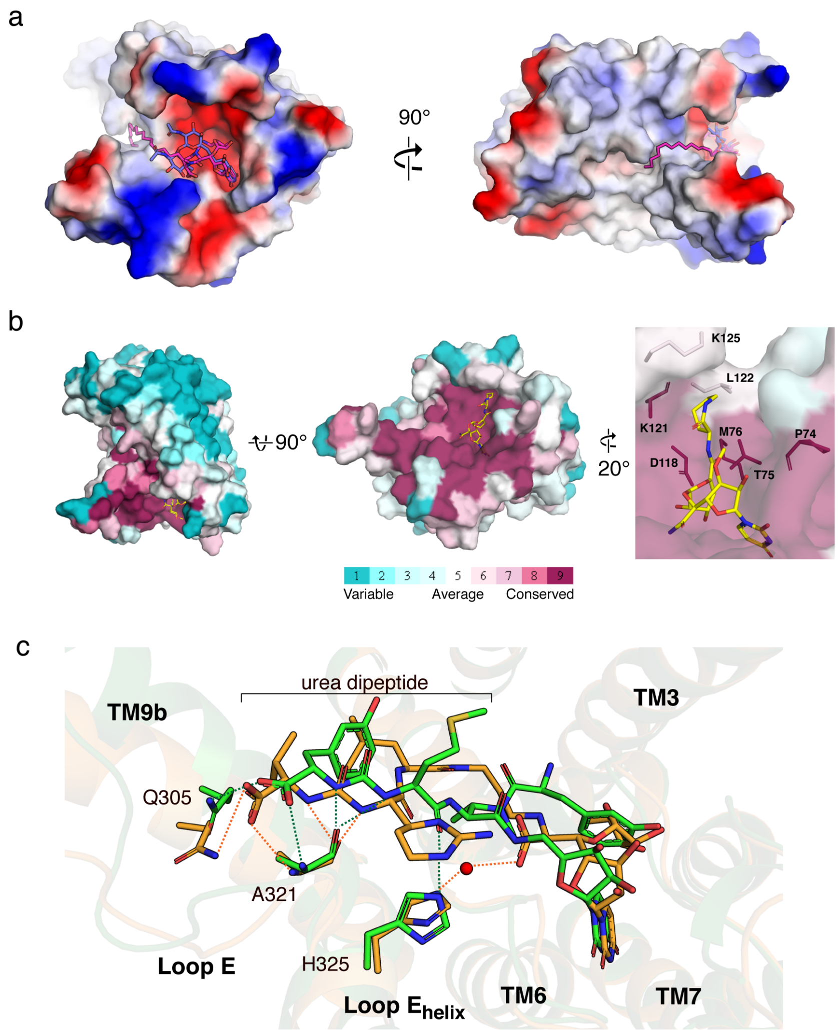

Supplementary Figure 6. Notable structural features of MraY$_{AA}$ in complex with carbacaprazamycin, capuramycin, and 3′-hydroxymureidomycin A. (a) Electrostatic surface representation of MraY$_{AA}$ in complex with carbacaprazamycin (magenta) and overlaid with tunicamycin (slate), as viewed from the cytoplasm (left) and in the plane of the membrane (right). (b) The structure of the MraY$_{AA}$-capuramycin complex, with increasing sequencing conservation mapped onto the MraY$_{AA}$ surface from cyan (least) to magenta (most). Capuramycin is shown as yellow sticks. Membrane (left), cytoplasm (middle), and caprolactam binding site (right) views of MraY$_{AA}$ are shown in surface representation. The side chain of K121 is disordered and could not be definitively modeled. Conservation was calculated using a sequence alignment of 30 MraY orthologs. (c) Structural overlay of MraY$_{AA}$ bound to 3′-hydroxymureidomycin A (green) and muraymycin D2 (orange; PDB ID: 5CKR). Hydrogen bonds formed between the urea dipeptide motif of each compound and the TM9b/Loop E pocket are shown in green and orange for 3′-hydroxymureidomycin A and muraymycin D2, respectively. A water molecule in the muraymycin D2-bound structure is represented as a red sphere.

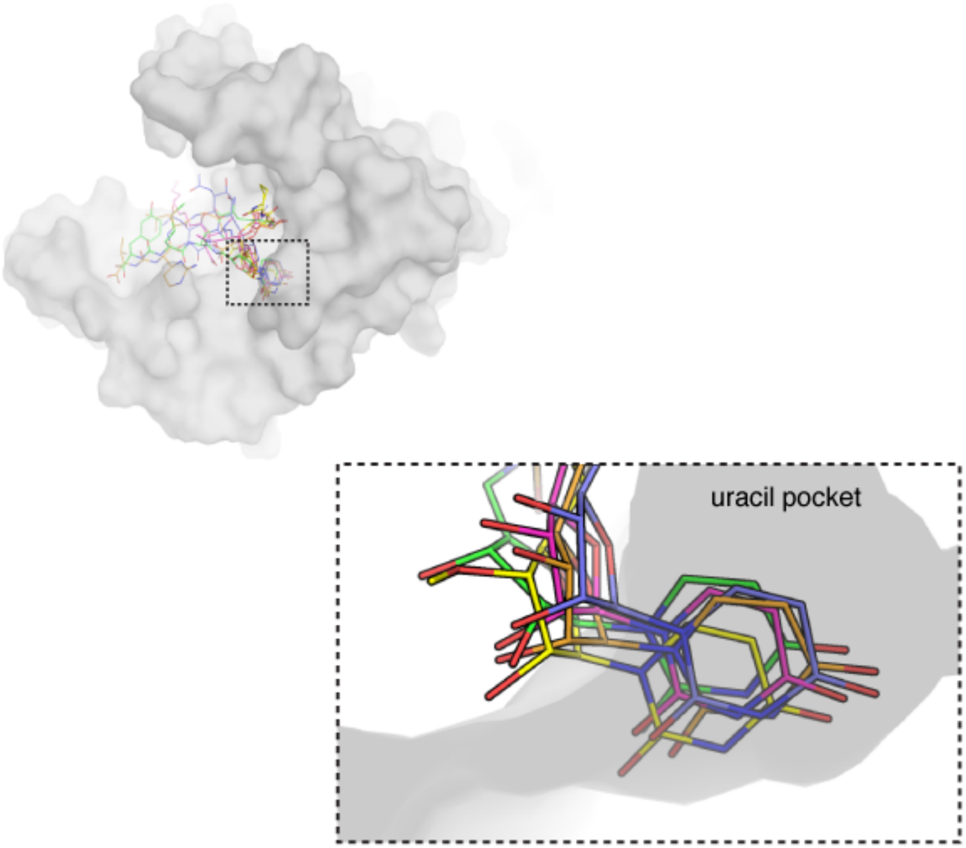

Supplementary Figure 7. Nucleoside inhibitors bind similarly to the uridine pocket of MraY. Structural superimposition of carbacaprazamycin (magenta), capuramycin (yellow), 3′-hydroxymureidomycin A (green), muraymycin D2 (orange), and tunicamycin (slate) with a zoomed-in view of the uridine binding pocket. The uracil moiety of each nucleoside inhibitor binds to an enclosed pocket on the cytoplasmic face of MraY, while the ribosyl moiety is more solvent exposed.

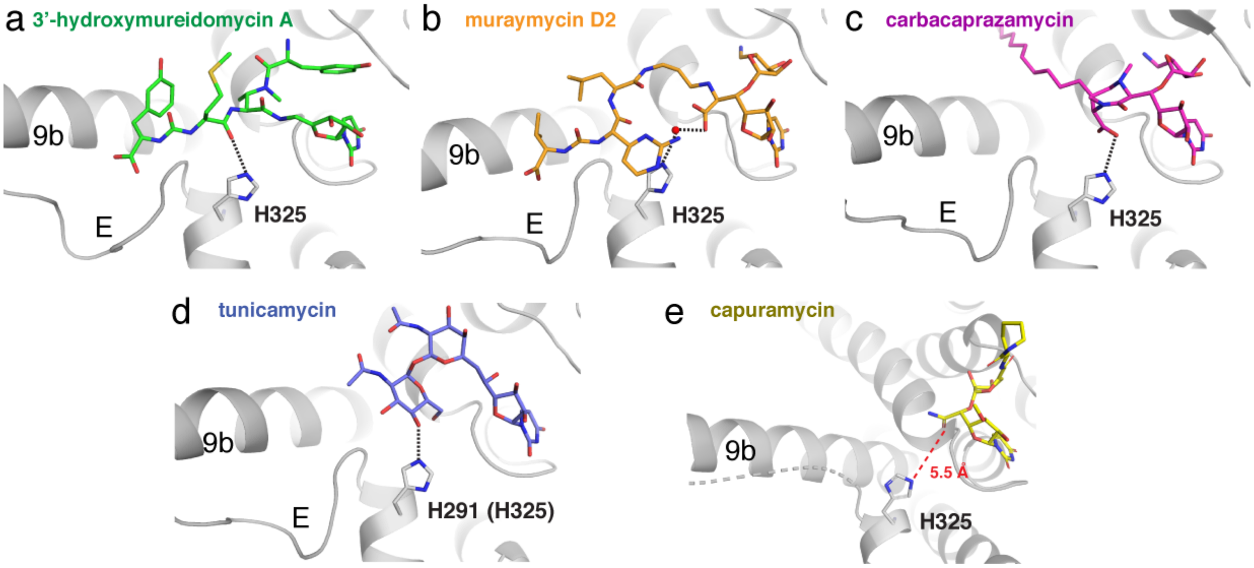

Supplementary Figure 8. Each inhibitor interacts with the Loop E helix except capuramycin. Interaction between H325 in $MraY_{AA}$ (H291 in $MraY_{CB}$) and (a) 3′-hydroxymureidomycin A, (b) muraymycin D2, (c) carbacaprazamycin, (d) tunicamycin, (e) capuramycin. Black dashed lines represent hydrogen bonds and the water molecule in (b) is shown as a red sphere. In the capuramycin-$MraY_{AA}$ complex structure (e), the amide moiety of capuramycin is too far to engage in a hydrogen bonding interaction with H325 (the distance between N4 and the carbonyl oxygen is represented by a red dashed line). Loop E in (e) is disordered and represented by a gray dashed line.

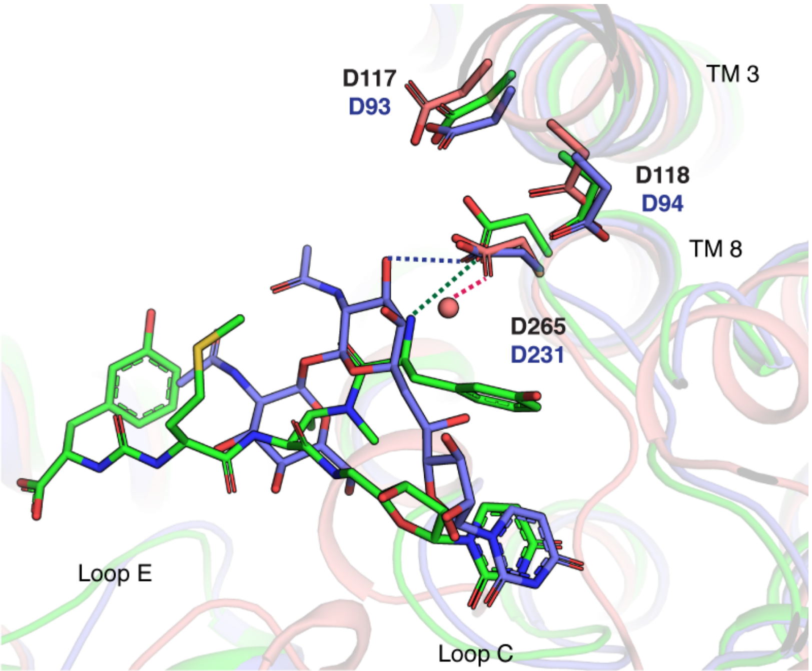

Supplementary Figure 9. Tunicamycin and 3′-hydroxymureidomycin A interact with the Mg^{2+} cofactor binding site in MraY. Structural overlay of $MraY_{AA}$ bound to 3′-hydroxymureidomycin A (green), $MraY_{CB}$ bound to tunicamycin (slate; PDB ID: 5JNQ), and $MraY_{AA}$ bound to Mg^{2+} (salmon; PDB ID: 4J72). Hydrogen bonds are represented by dashed lines and are color-coded according to the structures with which they are associated. Amino acid residue numbering is shown in black ($MraY_{AA}$) and in blue ($MraY_{CB}$).

Supplementary Methods

Synthesis of 3′-hydroxymureidomycin A

(*S*)-2-[(*tert*-Butoxycarbonyl)amino]-3-(3-hydroxyphenyl)propanoic acid (1)

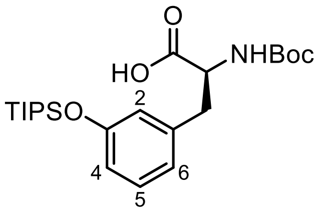

A mixture of *m*Tyr (1.50 g, 8.28 mmol) and $NaHCO_3$ (1.04 g, 12.4 mmol, 1.5 *eq*.) in THF (40 mL) and H_2O (40 mL) was treated with Boc_2O (1.99 g, 9.11 mmol, 1.1 *eq*.) at 0 ºC, and the mixture was stirred at room temperature for 17 h. The mixture was partitioned between EtOAc and 1 M *aq*. HCl. The organic phase was washed with brine, dried over Na_2SO_4, filtered and concentrated *in vacuo* to afford a crude carboxylic acid. A solution of the crude carboxylic acid and imidazole (3.38 g, 49.7 mmol, 6.0 *eq*.) in CH_2Cl_2 (83 mL) was treated with TIPSCl (5.26 mL, 24.8 mmol, 3.0 *eq*.) at 0 ºC, and the mixture was stirred at room temperature for 44 h. The mixture was partitioned between EtOAc and 1 M *aq*. HCl. The organic phase was washed with water and brine, dried over Na_2SO_4, filtered and concentrated *in vacuo*. A solution of the residue in THF/MeOH (2/1, 83 mL) and H_2O (13 mL) was treated with K_2CO_3 (1.60 g, 11.6 mmol, 1.4 *eq*.) at room temperature for 2 h. The mixture was partitioned between EtOAc and 1 M *aq*. HCl, and the organic phase was washed with water and brine, dried over Na_2SO_4, filtered and concentrated *in vacuo*. The residue was purified by silica gel column chromatography (50% EtOAc/hexane) to afford **1** (3.30 g, 7.54 mmol, 91%) as a colorless oil.

Data for **1**: ^{1}H NMR ($CDCl_3$, 500 MHz) δ 7.15 (dd, 1H, H-5-*m*Tyr, $J_{5\text{-}m\text{Tyr, }4\text{-}m\text{Tyr}} = J_{5\text{-}m\text{Tyr, }6\text{-}m\text{Tyr}}$ = 7.7 Hz), 6.80-6.68 (m, 3H, H-2-*m*Tyr, H-4-*m*Tyr, H-6-*m*Tyr), 4.89 (br d, 1H, N*H*, $J_{\text{N}H\text{, }\alpha\text{-}m\text{Tyr}}$ = 7.4 Hz), 4.57 (m, 1H, H-α-*m*Tyr), 3.12 (dd, 1H, H-β-*m*Tyr, $J_{\beta\text{-}m\text{Tyr, }\alpha\text{-}m\text{Tyr}}$ = 4.6, J_{gem} = 13.7 Hz), 3.03 (dd, 1H, H-β-*m*Tyr, $J_{\beta\text{-}m\text{Tyr, }\alpha\text{-}m\text{Tyr}}$ = 6.3, J_{gem} = 13.7 Hz), 1.42 (s, 9H, ^{t}Bu), 1.24 (m, 3H, Si[C*H*(CH_3)$_2$]$_3$), 1.10 (s, 12H, Si[CH(C*H*$_3$)$_2$]$_3$), 1.08 (s, 6H, Si[CH(C*H*$_3$)$_2$]$_3$); ^{13}C NMR ($CDCl_3$,

400 MHz) δ 176.8, 156.3, 137.3, 129.7, 122.1, 121.2, 118.8, 80.4, 54.3, 37.6, 28.4, 18.1, 12.8; ESIMS-LR *m/z* 438 [$(M + H)^+$] ; ESIMS-HR calcd for $C_{23}H_{39}N_1O_5SiNa$ 460.2490, found 460.2503.

tert-Butyl(_S_)-2-[(_tert_-butoxycarbonyl)amino]-3-{3-[(triisopropylsilyl)oxy]phenyl}propanoate (2)

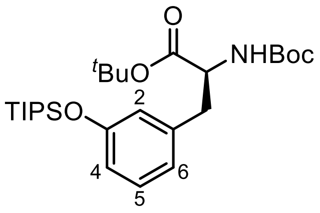

A solution of **1** (2.51 g, 5.74 mmol) in CH_2Cl_2 (57 mL) was treated with *O*-*tert*-butyl 2,2,2-trichloroacetmidate (5.14 mL, 28.7 mmol, 5.0 *eq*.) at room temperature. The mixture was warmed to 45 ºC and stirred for 2 h. Additional *O*-*tert*-butyl 2,2,2-trichloroacetmidate (5.14 mL, 28.7 mmol, 5.0 *eq*.) was added to the mixture, and the mixture was stirred for 2 h. The mixture was concentrated *in vacuo*. The residue was purified by silica gel column chromatography (3% EtOAc/hexane) to afford **2** (2.70 g, 5.47 mmol, 95%) as a colorless oil.

Data for **2**: ^{1}H NMR ($CDCl_3$, 400 MHz) δ 7.11 (dd, 1H, H-5-*m*Tyr, $J_{5\text{-}m\text{Tyr},\,4\text{-}m\text{Tyr}} = J_{5\text{-}m\text{Tyr},\,6\text{-}m\text{Tyr}}$ = 8.0 Hz), 6.75-6.70 (m, 3H, H-2-*m*Tyr, H-4-*m*Tyr, H-6-*m*Tyr), 4.96 (br d, 1H, N*H*, $J_{\text{N}H,\,\alpha\text{-}m\text{Tyr}}$ = 8.2 Hz,), 4.43 (dd, 1H, H-α-*m*Tyr, $J_{\alpha\text{-}m\text{Tyr},\,\text{N}H}$ = 8.2, $J_{\alpha\text{-}m\text{Tyr},\,\beta\text{-}m\text{Tyr}}$ = 6.0 Hz), 3.00 (d, 2H, H-β-*m*Tyr, $J_{\beta\text{-}m\text{Tyr},\,\alpha\text{-}m\text{Tyr}}$ = 6.0 Hz), 1.42 (s, 9H, Boc-^{t}Bu), 1.41 (s, 9H, ^{t}Bu), 1.29-1.19 (m, 3H, Si[C*H*$(CH_3)_2]_3$), 1.10 (d, 18H, *J* = 6.9 Hz, Si[CH(C$H_3)_2]_3$); ^{13}C NMR ($CDCl_3$, 400 MHz) δ 171.0, 156.1, 155.2, 137.8, 129.3, 122.4, 121.4, 118.3, 82.1, 79.7, 54.8, 38.3, 28.5, 28.1, 18.1, 12.8; ESIMS-LR *m/z* 494 [$(M + H)^+$].

***tert*-Butyl (*S*)-2-amino-3-{3-[(triisopropylsilyl)oxy]phenyl)}propanoate trifluoroacetate salt (3)**

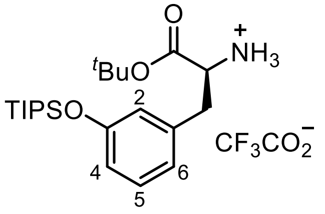

Compound **2** (2.60 g, 5.27 mmol) was treated with 10% TFA/CH_2Cl_2 (53 mL) at room temperature for 1.5 h. The mixture was concentrated *in vacuo*. The residue was purified by silica gel column chromatography (3% MeOH/$CHCl_3$ containing 0.1% TFA) to afford **3** (2.60 g, 5.30 mmol, 97%) as a colorless oil.

Data for **3**: ^{1}H NMR (CD_3OD, 400 MHz) δ 7.24 (dd, 1H, H-5-*m*Tyr, $J_{5\text{-}m\text{Tyr, }4\text{-}m\text{Tyr}} = J_{5\text{-}m\text{Tyr, }6\text{-}m\text{Tyr}}$ = 7.8 Hz), 6.88-6.80 (m, 3H, H-2-*m*Tyr, H-4-*m*Tyr, H-6-*m*Tyr), 4.12 (dd, 1H, H-α-*m*Tyr, $J_{\alpha\text{-}m\text{Tyr, }\beta\text{-}m\text{Tyr}}$ = 7.8, $J_{\alpha\text{-}m\text{Tyr, }\beta\text{-}m\text{Tyr}}$ = 6.9 Hz), 3.11 (m, 2H, H-β-*m*Tyr), 1.42 (s, 9H, ^{t}Bu), 1.29 (m, 3H, Si[C*H*(CH_3)$_2$]$_3$), 1.13 (s, 12H, Si[C*H*(CH_3)$_2$]$_3$), 1.11 (s, 6H, CH(C*H*$_3$)$_2$); ESIMS-LR *m/z* 394 [(M + H)$^+$]. This is a known, reported compound[1].

Ureadipeptide 4

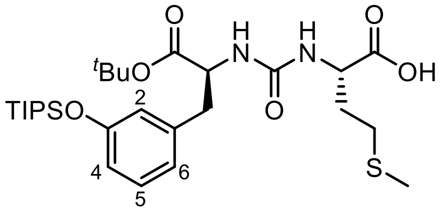

2-Chlorotrityl chloride resins (1.6 mmol/g, 200 mg) were washed with CH_2Cl_2 (2 mL), and a solution of Fmoc-Met-OH (356 mg, 960 μmol, 3.0 *eq.*) and ^{i}Pr$_2$NEt (334 μL,1.92 mmol, 6.0 *eq.*) in CH_2Cl_2 (2 mL) was then added. The mixture was agitated for 3 h at room temperature. Solvent and reagents were removed by suction. All resins were subjected to the following washing treatments with CH_2Cl_2/MeOH/^{i}Pr$_2$NEt (17/2/1, 2 mL × 3), DMF (2 mL × 2) and CH_2Cl_2 (2 mL × 2). All resins were treated with piperidine/DMF (1/4, 5 min, then 1/9, 15 min) to remove the Fmoc group at room temperature and washed with DMF (2 mL × 3) and CH_2Cl_2 (2 mL × 3). A solution of CDI

(156 mg, 960 μmol, 3.0 *eq.*) and NMM (211 mg, 1.92 μmol, 6.0 *eq.*) in CH_2Cl_2 (2 mL) was added to each resin. The mixture was agitated for 1 min at room temperature, and solvent and reagents were removed by suction. This procedure was repeated for 5 times. All resins were washed with CH_2Cl_2 (2 mL × 3). Then, a solution of H-*m*Tyr(TIPS)-O^{t}Bu (189 mg, 480 μmol, 1.5 *eq.*) and ^{i}Pr$_2$NEt (167 μL, 960 μmol, 3.0 *eq.*) in DMF (2 mL) was added to the resins. The mixture was agitated for 3 h at room temperature, and solvent and reagents were removed by suction. All resins were washed with DMF (2 mL × 3) and CH_2Cl_2 (2 mL × 3). Next, all resins were treated with 1% TFA/CH_2Cl_2 to cleaved from the resin, and the filtrate was patitioned between EtOAc and H_2O. The organic phase was washed with brine, dried, filtrated and concentrated *in vacuo* to afford **4** (141 mg, 248 μmol, 78 %) as a colorless oil.

Data for **4**: ^{1}H NMR (CD_3OD, 400 MHz) δ 7.14 (dd, 1H, H-5-*m*Tyr, $J_{5\text{-}m\text{Tyr, }4\text{-}m\text{Tyr}} = J_{5\text{-}m\text{Tyr, }6\text{-}m\text{Tyr}}$ = 7.9 Hz), 6.82 (d, 1H, H-6-*m*Tyr, $J_{6\text{-}m\text{Tyr, }5\text{-}m\text{Tyr}}$ = 7.9 Hz), 6.76-6.73 (m, 2H, H-4-*m*Tyr, H-2-*m*Tyr), 4.42-4.38 (m, 2H, H-α-*m*Tyr,, H-α-Met), 2.95 (d, 2H, H-β- *m*Tyr, $J_{\beta\text{-}m\text{Tyr, }\alpha\text{-}m\text{Tyr}}$ = 6.8 Hz), 2.56-2.52 (m, 2H, H-γ-Met) 2.12-2.08 (m, 1H, H-β-Met), 2.08 (s, 3H, SC*H$_3$*), 1.94-1.84 (m, 1H, H-β-Met), 1.40 (s, 9H, ^{t}Bu), 1.28-1.19 (m, 3H, Si[C*H*(CH$_3$)$_2$]$_3$),1.11 (d, 18H, Si[CH(C*H$_3$*)$_2$]$_3$, *J* = 7.3 Hz); ^{13}C NMR (CD_3OD, 100 MHz) δ 176.8, 173.1, 159.7, 157.3, 139.7, 130.3, 123.6, 122.1, 119.1, 82.8, 56.2. 53.2, 39.3, 33.4, 31.0, 28.3, 18.4, 15.3, 13.9; ESIMS-HR calcd for $C_{28}H_{49}N_2O_6SSi_2$ 569.3075, found 569.3064.

Compound 5

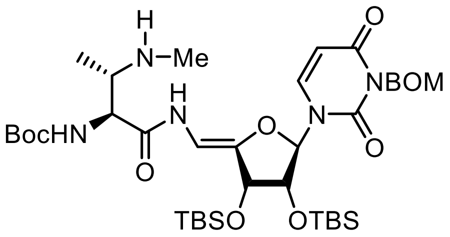

A solution of (*2S,3S*)-3-((benzyloxy)methylamino)-2-(tert-butoxyformamido)butyric acid[2] (2.55 g, 6.78mmol) in

MeOH (68 mL) was added by $Pd(OH)_2$ (1.1 g) at room temperature. The mixture was vigorously stirred for 3 h under H_2 atmosphere. The catalyst was filtered off through a celite pad, and the filtrare was concentrated *in vacuo*. The crude carboxamide, 3-(benzyloxymethyl)-1-[2,3-di-O-(tert-butyldimethylsilyl)-4,5-dehydro-5-iodo-β-D-ribo-pentofuranosyl]uracil[3] (4.75g, 6.78 mmol ,1.0 *eq*.), CuI (1.25 g, 8.14 mmol, 1.2 *eq*.) and Cs_2CO_3 (13.1 g, 10.2 mmol, 1.5 *eq*.) were added to flask. Dried THF (68 mL) and *N*,*N*-dimethyl-1,2-ethylendiamine (2.8 mL, 16.3 mmol, 2.4 *eq*.) were added to the mixture, which was heated on 70 °C for 7 h. The reaction mixture diluted with EtOAc, and the insoluble substances were filtered off through a celite pad, and the filtrare was washed with H_2O and brine, dried over Na_2SO_4, filtrated, and concentrated *in vacuo*. The residue was purified by silica gel column chromatography ($CHCl_3$/MeOH = 6-10 %) to afford **5** (3.65 g, 4.53 mmol, 67 %) as a white foam.

Data for **5**: ^1H NMR ($CDCl_3$, 400 MHz) δ 9.15 (br s, 1H, NH), 7.38-7.27 (m, 5H, $NCH_2OCH_2C_6H_5$), 7.17 (d, 1H, H-6, $J_{6,5}$ = 8.2 Hz), 6.30 (s, 1H, H-5′), 6.29 (d, 1H, H-1′, $J_{1',2'}$ = 6.0 Hz), 5.86 (d, 1H, H-5, $J_{5,6}$ = 8.2 Hz), 5.50 (d, 2H, NCH_2OBn, J_{gem} = 6.4 Hz), 4.69 (s, 2H, NCH_2OCH_2Ph), 4.40 (d, 1H, H-3′, $J_{3',2'}$ = 4.1 Hz), 4.17 (dd, 1H, H-2′, $J_{2',1'}$ = 6.0, $J_{2',3'}$ =4.1 Hz), 4.05 (dd, 1H, H-α-DABA, $J_{\alpha\text{-DABA},\beta\text{-DABA}}$ = 6.9 Hz), 2.74 (dq, 1H, H-β-DABA, $J_{\beta\text{-DABA},\alpha\text{-DABA}}$ = 6.9, $J_{\beta\text{-DABA},\gamma\text{-DABA}}$ = 6.4 Hz), 2.38 (s, 3H, NCH_3), 1.41 (s, 9H, Boc), 1.13 (d, 3H, H-γ-DABA, $J_{\gamma\text{-DABA},\beta\text{-DABA}}$ = 6.4 Hz), 0.90, 0.83 (s, 18H, $(CH_3)_3CSiMe_2$ × 2), 0.11, 0.09, 0.03, −0.03 (s, 12H, $^tBuCSi(CH_3)_2$ × 4); ^{13}C NMR ($CDCl_3$, 100 MHz) δ 168.4, 162.4, 156.5, 151.2, 141.4, 138.3, 137.8, 128.4, 127.8, 103.2, 100.9, 89.6, 75.8, 72.2, 71.2, 70.5, 33.8, 28.4, 25.9, 25.7, 18.3, 18.0, 16.2, −4.1, −4.4, −4.9; ESIMS-LR *m*/*z* 804.99, [$(M+H)^+$]; ESIMS-HR calcd for $C_{39}H_{66}N_5O_9Si_2$ 804.4394, found 804.4399; $[\alpha]^{19}_D$ −28.1 (*c* 0.75, $CHCl_3$).

Compound 6

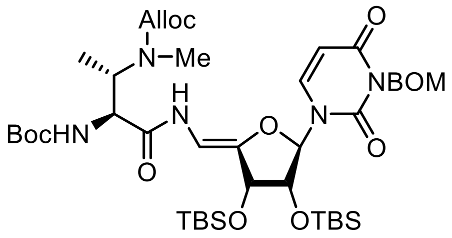

A solution of **5** (2.62 g, 3.25 mmol) and AllocCl (414.8 μmol, 3.90 mmol, 1.2 *eq*.) in THF/*sat. aq*. $NaHCO_3$ (1/1, 40 mL) was stirred at 0 °C for 5 h. The reaction mixture was quenched with MeOH, and the organic solvents were removed under reduced pressure. The resulting aqueous phase was extracted with EtOAc, which was washed with H_2O and brine, dried over Na_2SO_4, filtrated and concentrated *in vacuo*. The residue was purified by silica gel column chromatography (ϕ 5 × 10 cm, hexane/EtOAc = 10-30%) to afford **6** (2.7 mg, 3.0 μmol, 92%) as a white foam.

Date for **6**: ^{1}H NMR (CD_3OD, 500 MHz, 1:1 mixture of rotamers) δ 7.70, 7.68 (each d, 1H, H-6, $J_{6,5}$ = 8.0, 8.0 Hz), 7.33-7.28 (m, 5H, $NCH_2OCH_2C_6H_5$), 6.40, 6.35 (each d, 1H, H-1′, $J_{1',2'}$ = 6.3, 6.9 Hz), 6.25 (s, 1H, H-5′), 5.97-5.88 (m, 2H, H-5, C*H*-Alloc), 5.48 (s, 5H, C*H*$_2$OBn), 5.31, 5.29 (each d, 1H, C*H*-Alloc, *J* = 17.2, 16.6 Hz), 5.18 (d, 1H, C*H*-Alloc, *J* = 10.3 Hz), 4.65 (s, 2H, NCH_2OCH_2Ph), 4.58-4.51 (m, 4H, H-2′, H-3′, C*H*$_2$-Alloc), 4.44, 4.28 (each m, 1H, H-α-DABA,), 4.37 (m, 1H, H-β-DABA), 2.81 (s, 3H, NC*H*$_3$), 1.42 (s, 9H, Boc), 1.21 (m, 3H, H-γ-DABA), 0.94, 0.85 (s, 18H, $(CH_3)_3CSiMe_2$ × 2), 0.17, 0.14, 0.08, −0.02 (s, 12H, ^{t}BuCSi$(CH_3)_2$ × 4); ^{13}C NMR (CD_3OD, 125 MHz, 1:1 mixture of rotamers) δ 169.7, 164.2, 157.6, 152.8, 144.0, 141.4, 141.2, 139.1, 134.2, 129.3, 128.8, 128.7, 117.9, 117.6, 1103.7, 103.5, 101.4, 101.3, 91.0, 90.3, 81.0, 76.5, 76.1, 73.0, 72.7, 71.5, 67.6, 67.3, 58.4, 54.5, 30.4, 28.7, 26.4, 26.2, 19.1, 18.8, 15.2, 14.3, −3.9. −4.2, −4.3, −4.8; ESIMS-LR *m*/*z* 888.44, $[(M+H)^+]$; ESIMS-HR calcd for $C_{43}H_{70}N_5O_{11}Si_2$ 888.4605, found 888.4598; $[\alpha]^{24}_D$ −0.65 (*c* 1.06, $CHCl_3$).

Compound 7

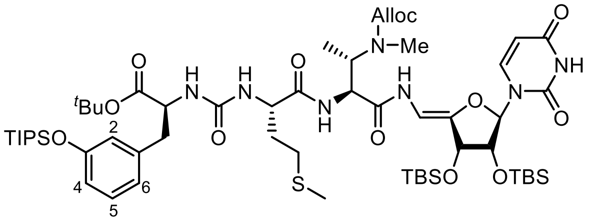

A solution of **6** (444.1 mg, 500 μmol) in CH_2Cl_2 (10 mL) was treated with 1 M BCl_3 in CH_2Cl_2 (5 mL, 5 mmol, 10.0 *eq*.) at −78 °C for 30 min followed by at 0 °C for 15 min. After *sat. aq*. $NaHCO_3$ was added to the mixture, the resulting biphasic mixture was stirred for 15 min. The mixture was extracted with CH_2Cl_2, which was washed with *sat. aq*. $NaHCO_3$, H_2O and brine. The organic layer was dried over Na_2SO_4, filtrated and concentrated *in vacuo*. The residue was purified by silica gel column chromatography (ϕ 2 × 10 cm, $CHCl_3$/MeOH = 0-1-2-5%) to afford a crude amine (177.9 mg, 266.3 μmol, 53%) as a white foam. A mixture of the crude amine (47.3 mg, 70.7 μmol), ureadipeptide **4** (60.4 mg, 106.1 μmol, 1.5 *eq*.), HOBt (14.3 mg, 106.1 μmol, 1.5 *eq*.) and ^{i}Pr$_2$NEt (37.0 μL, 212.2 μmol, 3.0 *eq*.) in CH_2Cl_2 (1 mL) was treated with EDCI (20.4 mg, 106.1 mmol, 1.5 *eq*.) at room temperature for 12 h. The reaction mixture was diluted with EtOAc and washed with 0.1 M *aq*. HCl, *sat. aq*. $NaHCO_3$, H_2O and brine. The organic layer was dried over Na_2SO_4, filtrated and concentrated *in vacuo*. The residue was purified by flash silica gel column chromatography (ϕ 1.5 × 10 cm, $CHCl_3$/MeOH = 0-1-2%) to afford **7** (64.2 mg, 53 μmol, 75%) as a colorless amorphous solid.

Data for **7**: ^{1}H NMR (CD_3OD, 500 MHz, 1:1 mixture of rotamers) δ 7.68, 7.61 (each d, 1H, H-6, $J_{6,5}$ = 8.0, 8.0 Hz), 7.14 (dd, 1H, H-5-*m*Tyr, $J_{5\text{-}m\mathrm{Tyr},\,4\text{-}m\mathrm{Tyr}} = J_{5\text{-}m\mathrm{Tyr},\,6\text{-}m\mathrm{Tyr}}$ = 7.6 Hz), 6.80 (d, 1H, H-6-*m*Tyr, $J_{6\text{-}m\mathrm{Tyr},\,5\text{-}m\mathrm{Tyr}}$ = 7.6 Hz), 6.74 (s, 1H, H-2-*m*Tyr), 6.74 (d, 1H, H-4-*m*Tyr, $J_{4\text{-}m\mathrm{Tyr},\,5\text{-}m\mathrm{Tyr}}$ =7.6 Hz), 6.38, 6.33 (each d, 1H, H-1′, $J_{1',2'}$ = 6.9, 6.9 Hz), 6.26, 6.24 (each s, 1H, H-5′), 5.94 (ddt, 1H, C*H*-Alloc, *J* = 16.6, 10.9, 5.7 Hz), 5.86, 5.80 (each dd, 1H, H-5, $J_{5,6}$ = 8.0, 8.0 Hz), 5.31, 5.28 (each d, 1H, C*H*-Alloc, *J* = 16.6 Hz), 5.18 (d, 1H, C*H*-Alloc, *J* = 10.9 Hz), 4.71, 4.63

(each d, 1H, H-α-DABA, $J_{\alpha\text{-DABA}, \beta\text{-DABA}}$ = 8.0, 10.3 Hz), 4.58-4.52(m, 3H, H-3′, C*H₂*-Alloc), 4.48-4.37 (m, 2H, H-2′, H-β-DABA), 4.41 (t, 1H, H-α-*m*Tyr, $J_{\alpha\text{-}m\text{Tyr}, \beta\text{-}m\text{Tyr}}$ = 6.9 Hz), 4.35 (m, 1H, H-α-Met) , 2.92 (d, 2H, H-β-*m*Tyr, $J_{\beta\text{-}m\text{Tyr}, \alpha\text{-}m\text{Tyr}}$ = 6.9Hz), 2.81, 2.80 (s, 3H, NCH_3), 2.47 (m, 2H, H-γ-Met), 2.04 (s, 3H, SCH_3-Met), 1.98 (dddd, 1H, H-β-Met, J_{gem} = $J_{\beta\text{-Met}, \alpha\text{-Met}}$ = 14.2, $J_{\beta\text{-Met}, \gamma\text{-Met}}$ = 6.9, 2.3 Hz), 1.83 (ddd, 1H, H-β-Met, J_{gem} = $J_{\beta\text{-Met}, \alpha\text{-Met}}$ = 14.2, $J_{\beta\text{-Met}, \gamma\text{-Met}}$ = 8.0 Hz), 1.39 (s, 9H, ^{t}Bu), 1.29-1.24 (m, 3H, Si[C*H*(CH_3)$_2$]$_3$), 1.21 (d, 3H, H-γ-DABA, $J_{\gamma\text{-DABA}, \beta\text{-DABA}}$ = 6.9 Hz), 1.10 (d, 18H, Si[CH(C*H₃*)$_2$]$_3$, *J* = 7.5 Hz), 0.92, 0.88 (each s, each 9H, (C*H₃*)$_3$CSiMe$_2$ × 2), 0.15, 0.12, 0.08, 0.01(each s, each 3H, ^{t}BuCSi(C*H₃*)$_2$ × 4); ^{13}C NMR (CD_3OD, 125 MHz, 1:1 mixture of rotamers) δ 174.6, 173.0, 169.1, 168.9, 165.5, 159.4, 157.3, 152.3, 152.3, 144.1, 144.0, 142.1, 139.7, 134.3, 130.3, 123.5, 122.1, 119.0, 118.0, 117.6, 104.0, 101.3, 89.3, 82.8, 76.6, 76.3, 72.7, 67.6, 67.3, 56.7, 56.2, 54.6, 54.1, 39.4, 33.7, 30.9, 30.5, 28.3, 27.9, 26.3, 26.2, 19.0, 18.8, 18.5, 15.4, 14.5, 13.9 ,−3.9, −4.2, −4.3, −4.9; ESIMS-LR *m*/*z* 1218.66, [(M+H)$^+$]; ESIMS-HR calcd for $C_{58}H_{100}N_7O_{13}SSi_3$ 1218.6402, found 1218.6417; $[\alpha]^{23}_D$ +15.5 (*c* 0.89, $CHCl_3$).

Compound 8

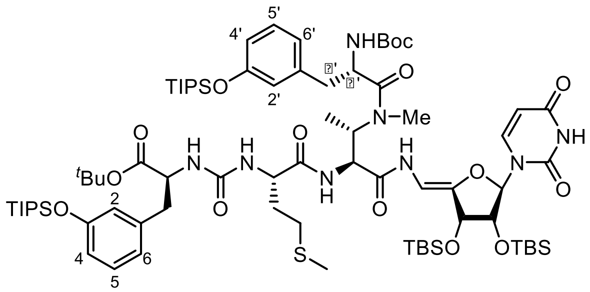

A solution of **7** (44.6 mg, 36.6 μmol), morpholine (12.6 μL, 146.4 μmol, 4.0 *eq*.) in THF (400 μL) was treated with $Pd(PPh_3)_4$ (21.1 mg, 18.3 μmol, 0.5 *eq*.) at room temperature for 2 h. Aafter SH silica gel was added to the mixture, the whole was stirred for 1 h, and the SH silica gel was filtered off. The filtrate was concentrated *in vacuo*. A mixture of the crude amine, Boc-*m*Tyr(TIPS)-OH (32.0 mg, 73.2 μmol, 2.0 *eq*.) and ^{i}Pr$_2$NEt (25.6 μL, 146.4 μmol, 4.0 *eq*.) in

DMF (400 μL) was treated with HATU (27.8 mg, 73.2 μmol, 2.0 *eq*.) at room temperature for 5 h. The reaction mixture was diluted with EtOAc/hexane (4/1), which was washed with 0.1 M *aq*. HCl, *sat. aq*. $NaHCO_3$, H_2O and brine. The organic layer was dried over Na_2SO_4, filtrated and concentrated *in vacuo*. The residue was purified by flash silica gel column chromatography (ϕ 1 × 10 cm, $CHCl_3$/MeOH = 0-1-2-5%, then hexane/EtOAc = 50%) to afford **8** (24.8 mg, 16.0 μmol, 44%) as colorless amorphous solid.

Data for **8**: ^{1}H NMR (CD_3OD, 500 MHz, 3:2 mixture of rotamers, selected date for the major rotamer) δ 7.38 (d, 1H, H-6, $J_{6,5}$ = 8.0 Hz), 7.18-7.11 (m, 2H, H-5-*m*Tyr, H-5′-*m*Tyr), 6.88 (d, 1H, H-4′-*m*Tyr, $J_{4'\text{-}m\text{Tyr},\, 5'\text{-}m\text{Tyr}}$ = 7.5 Hz), 6.83 (br s, 1H, H-2′-*m*Tyr), 6.81-6.68 (m, 4H, H-2-*m*Tyr, H-4-*m*Tyr, H-6-*m*Tyr, H-6′-*m*Tyr), 6.49 (d, 1H, NH′-*m*Tyr, $J_{\text{NH}'\text{-}m\text{Tyr},\, \alpha'\text{-}m\text{Tyr}}$ = 8.6 Hz), 6.24 (s, 1H, H-5′), 6.19 (d, 1H, H-1′, $J_{1',2'}$ = 7.5 Hz), 5.73 (d, 1H, H-5, $J_{5,6}$ = 8.0 Hz), 4.91-4.84 (m, 1H, H-β-DABA), 4.71 (d, 1H, H-α-DABA, $J_{\alpha\text{-DABA},\, \beta\text{-DABA}}$ = 8.0 Hz), 4.68 (ddd, 1H, H-α′-*m*Tyr, $J_{\alpha'\text{-}m\text{Tyr},\, \beta'\text{-}m\text{Tyr}}$ = 10.3, $J_{\alpha'\text{-}m\text{Tyr},\, \text{NH}'\text{-}m\text{Tyr}}$ = 8.6, $J_{\alpha'\text{-}m\text{Tyr},\, \beta'\text{-}m\text{Tyr}}$ =3.4 Hz), 4.49 (d, 1H, H-3′, $J_{3',2'}$ = 4.0 Hz), 4.42-4.47 (m, 2H, H-2′, H-α-*m*Tyr), 4.37-4.30 (m, 1H, H-α-Met), 3.07 (s, 3H, NCH_3), 2.92 (d, 1H, H-β-*m*Tyr, $J_{\beta\text{-}m\text{Tyr},\, \alpha\text{-}m\text{Tyr}}$ = 6.9 Hz), 2.90-2.85 (m, 2H, H-β-*m*Tyr, H-β′-*m*Tyr), 2.59 (dd, 1H, H-β′-*m*Tyr, J_{gem} = 14.3, $J_{\beta'\text{-}m\text{Tyr},\, \alpha'\text{-}m\text{Tyr}}$ = 10.3 Hz), 2.51-2.45 (m, 2H, H-γ-Met), 2.05 (s, 3H, SCH_3-Met), 2.02-1.95 (m, 1H, H-β-Met), 1.87-1.80 (m, 1H, H-β-Met), 1.39 (s, 9H, ^{t}Bu), 1.34 (s, 9H, Boc), 1.31-1.20 (m, 9H, H-γ-DABA, $Si[CH(CH_3)_2]_3$ × 2), 1.12-1.10 (d, 36H, $Si[CH(CH_3)_2]_3$), 0.90, 0.85 (each s, each 9H, $(CH_3)_3CSiMe_2$ × 2), 0.12, 0.10, 0.01, −0.05 (each s, each 3H, ^{t}BuCSi$(CH_3)_2$ × 4); ^{13}C NMR (CD_3OD, 125 MHz, 1:1 mixture of rotamers) δ 174.8 174.7, 174.4, 174.0, 173.1, 173.0, 168.7, 165.6, 165.5, 159.5, 159.4, 157.6, 157.5, 157.4, 157.3, 115.2, 143.9, 143.7, 142.5, 140.4, 139.7, 139.7, 139.4, 130.7, 130.5, 130.4, 123.6, 123.4, 123.2, 122.2, 122.1,

122.1, 104.0, 101.5, 101.4, 91.0, 82.8, 80.9, 80.5, 76.5, 75.9, 72.9, 72.6, 56.9, 56.6, 56.2, 56.1, 55.8, 54.3, 53.7, 53.0, 52.3, 40.3, 39.5, 39.3, 38.4, 33.5, 33.3, 31.0, 30.8, 28.9, 28.7, 28.6, 28.3, 26.4, 26.3, 26.2, 19.1, 18.8, 18.5, 18.5, 18.2, 18.5, 15.4, 14.3, 14.2, 13.9, 13.7, −3.8, −4.1, −4.2, −4.9, −4.9; ESIMS-LR *m/z* 1554.62, $[(M+H)^+]$; ESIMS-HR calcd for $C_{77}H_{133}N_8O_{15}SSi_2$ 1553.8683, found 1553.8624; $[\alpha]^{20}_D$ +26.1 (*c* 0.70, $CHCl_3$).

3′-hydroxymureidomycin A

A solution of **8** (17.0 mg, 10.9 μmol) in MeCN (1 mL) was treated with $3HF\cdot Et_3N$ (71.3 μL, 437.5 μmol, 40.0 *eq.*) at room temterature for 50 h. The reaction mixture was concentrated and the residue was diluted with EtOAc. The organic layer was washed with *sat. aq.* $NaHCO_3$, H_2O and brine, dried over Na_2SO_4, filtered and concentrated *in vacuo*. The resulting residue was treated with 80% *aq.* TFA at room temperature for 2 h. After toluene was added, the mixture was concentrated *in vacuo*. The residue was washed with Et_2O, purified by ODS column chromatography (H_2O/MeCN = 0-30%, containing 0.1% TFA) to afford 3′-hydroxymureidomycin A (4.2 mg, 4.9 μmol, 45% over 2 steps) as a white powder after lyophilization.

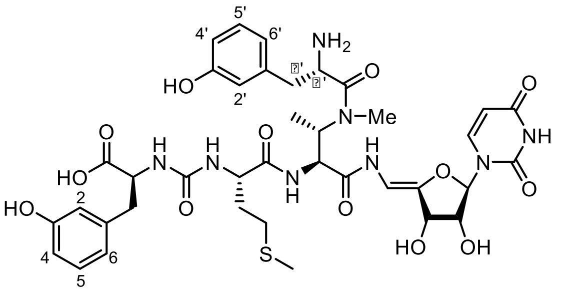

Data for 3′-hydroxymureidomycin A. ^{1}H NMR (D_2O, 500 MHz, 3:1 mixture of rotamers, selected date for the major rotamer) δ 7.30, 7.22 (each dd, each 1H, H-5-*m*Tyr, H-5′-*m*Tyr, $J_{5\text{-}m\mathrm{Tyr},\,6\text{-}m\mathrm{Tyr}} = J_{5'\text{-}m\mathrm{Tyr},\,6'\text{-}m\mathrm{Tyr}} = J_{5\text{-}m\mathrm{Tyr},\,4\text{-}m\mathrm{Tyr}} = J_{5\text{-}m\mathrm{Tyr},\,4'\text{-}m\mathrm{Tyr}}$ = 7.5 Hz), 7.10 (d, 1H, H-6, $J_{6,5}$ = 8.0 Hz), 6.87-6.73 (m, 6H, H-6-*m*Tyr, H-4-*m*Tyr, H-2-*m*Tyr, H-6′-*m*Tyr, H-4′-*m*Tyr, H-2′-*m*Tyr), 6.23 (s, 1H, H-5′), 6.06 (d, 1H, H-1′, $J_{1',2'}$ = 5.2 Hz), 5.65 (d, 1H, H-5, $J_{5,}$

$_6$ = 8.0 Hz), 4.95 (dq, 1H, H-β-DABA, $J_{\beta\text{-DABA}, \alpha\text{-DABA}}$ = 9.7, $J_{\beta\text{-DABA}, \gamma\text{-DABA}}$ = 6.9 Hz), 4.72 (d, 1H, H-3′, $J_{3', 2'}$ = 5.2 Hz), 4.63 (d, 1H, H-α-DABA, $J_{\alpha\text{-DABA}, \beta\text{-DABA}}$ = 9.2 Hz), 4.58 (dd, 1H, H-α′-*m*Tyr, $J_{\alpha'\text{-}m\text{Tyr}, \beta'\text{-}m\text{Tyr}}$ = 10.0, $J_{\alpha'\text{-}m\text{Tyr}, \beta'\text{-}m\text{Tyr}}$ = 4.0 Hz), 4.36 (dd, 1H, H-α-*m*Tyr, $J_{\alpha\text{-}m\text{Tyr}, \beta\text{-}m\text{Tyr}}$ = 8.3, $J_{\alpha\text{-}m\text{Tyr}, \beta\text{-}m\text{Tyr}}$ = 5.7 Hz), 4.27 (dd, 1H, H-α-Met, $J_{\alpha\text{-Met}, \beta\text{-Met}}$ = 8.0, $J_{\alpha\text{-Met}, \beta\text{-Met}}$ = 5.7 Hz), 4.24 (dd, 1H, H-2′, $J_{2', 3'}$ = $J_{2', 1'}$ = 5.2 Hz), 3.06 (s, 3H, NCH_3), 3.10-3.05 (m, 1H, H-β′-*m*Tyr), 3.02 (dd, 1H, H-β-*m*Tyr, J_{gem} = 14.3, $J_{\beta\text{-}m\text{Tyr}, \alpha\text{-}m\text{Tyr}}$ = 5.7 Hz), 2.88 (dd, 1H, H-β-*m*Tyr, J_{gem} = 14.2, $J_{\beta\text{-}m\text{Tyr}, \alpha\text{-}m\text{Tyr}}$ = 8.3 Hz), 2.73-2.66 (m, 1H, H-β′-*m*Tyr), 2.55-2.42 (m, 2H, H-γ-Met), 2.06 (s, 3H, SCH_3-Met), 2.00-1.95 (m, 2H, H-β-Met), 1.23 (d, 3H, H-γ-DABA, $J_{\gamma\text{-DABA}, \beta\text{-DABA}}$ = 6.9 Hz); ^{13}C NMR (D_2O, MeCN was used as an internal reference, apparent observed peaks are indicated because of the existence of multiple rotamers) δ 170.0, 163.6, 163.4, 156.0, 139.6, 135.8, 131.3, 130.5, 103.4, 99.8, 90.5, 72.9, 68.9, 55.6, 53.6, 53.5, 52.9, 38.1, 36.1, 31.3, 30.8, 29.9, 14.8, 13.5. ESIMS-LR *m/z* 857.12, [(M+H)$^+$]; ESIMS-HR calcd for $C_{38}H_{49}N_8O_{13}S$ 857.3134, Found 857.3122; $[\alpha]^{19}_D$ –-13.4 (*c* 0.15, H_2O).

References

1 White, J. D. & Suttisintong, K. Synthesis of the tripeptide domain of sanglifehrins using asymmetric phase-transfer catalysis. *J Org Chem* **78**, 2757-2762 (2013).

2 Boojamra, C. G. *et al.* Stereochemical elucidation and total synthesis of dihydropacidamycin D, a semisynthetic pacidamycin. *J Am Chem Soc* **123**, 870-874 (2001).

3 Okamoto, K. *et al.* Total synthesis of pacidamycin D by Cu(I)-catalyzed oxy enamide formation. *Org Lett* **13**, 5240-5243 (2011).
